# From discourse to practice: the circulation of norms, ideas and practices of migration management through the implementation of the mobility partnerships in Moldova and Georgia

**DOI:** 10.1186/s40878-017-0066-y

**Published:** 2018-03-23

**Authors:** Martine Brouillette

**Affiliations:** Migrinter Laboratory, University of Poitiers, Maison des Sciences de l’Homme et de la Société, Bât. A5, 5 rue Théodore Lefebvre, 86073 Poitiers Cedex 9, France

**Keywords:** Mobility partnership, Global approach to migration and mobility, External dimension of EU’s migration policies, Eastern Neighbourhood, Georgia, Moldova, Migration management, Europeanization, Policy instrument

## Abstract

This research wishes to contribute to the understanding of the migration policy regime of the European Union (EU), by considering an analytical perspective that privileges the standpoint of the countries of its neighbourhood. As an entry point, we have focused our analysis on the Mobility Partnership, a policy instrument of soft power, representative of the emblematic network governance privileged by the EU in its current political framework, the Global Approach to Migration and Mobility (GAMM). Applying an “instrument approach”, our research raises the question of the role played by the Mobility Partnership in the circulation of norms, ideas and practices related to the “good governance” of international migration, and whether these are internalized by the partner third countries. We present the results of a comparative analysis of two study-cases, Moldova and Georgia, countries considered by the European Commission as the “best pupils” in the implementation of their Mobility Partnerships, with the ambition to interrogate whether this instrument leads to a “common understanding” between the EU and the national actors that may lead to a translation of the European objectives in the field of migration into the registries of practices in the countries of the Eastern neighbourhood. Lastly, we will discuss the strategic “usage” of this instrument from the partner third countries, that can lead to different results, from complete absorption of the objectives, to resistance in their implementation.

## Introduction

According to the European Commission, the Global Approach to Migration, which came into force in 2005, represents the transition from a previously security-oriented migration policy to a more transparent and exhaustive strategy, that would be driven by a better understanding of all aspects related to migration, and considering mobility as a positive force for development (European Commission, 2008). This approach, defined by the European Commission as the external dimension of the European Union’s (EU) migration policy, contributes to the relocation of the attention paid by the EU in the field of migration management to the beginning of the migratory chain.

Under the framework of the Global Approach to Migration, the main instrument of cooperation with third countries is the Mobility Partnership. Taking the form of a political declaration between the partner third country, the European Commission and the participating member states, the Mobility Partnership is presented as an instrument elaborated to better manage circulation between the EU and the partner countries, by addressing the facilitation of legal migration, the fight against irregular migration, the enhancement of the link between migration and development and the development of the external dimension of asylum.

Previous analyses on the external dimension of EU’s migration policy often refer to third countries as the “fields of the externalization of European policies” (Guiraudon & Lahav, 2000; Guild, Carrera, & Balzacq, 2008; Bigo & Guild, 2010), implying that the EU is exporting its internal affairs concerns into its foreign relations with the countries of its neighbourhood. Over the years, the European Union has woven a web of relations with its neighbours through intersecting policy instruments, aiming at prescribing its norms of migration management, more specifically with regards to restrictive migration control. As Ian Manners has argued, the EU would be a normative power in the sense that it repeatedly attempts to “shape conceptions of the normal” through practices of “diffusion” of its best practices and its effort of capacity building in third countries (Manner, 2002). For some, the EU’s external action can be perceived as a form of “soft imperialism” (Longo, 2011), since even without resorting to “hard power”, the EU is able to impose its interest through its various platforms for dialogues and through the use of conditionality. The Mobility Partnership is embedded in the “global policy discourse” of migration management put forward by the EU, depoliticizing the issue of migration and adopting instead a more neutral technocratic mode of external governance (Kunz, Lavenex, & Panizzon, 2011) focused on the setting of rules, norms, procedures and best practices that become measurable through benchmarking methods or progress reports. As argued by Geiger and Pécoud, this performative discourse on what migration is and how it should be managed legitimizes the migration management activities and gradually transforms the way it is perceived by local actors, notably in the countries of origin (Geiger & Pécoud, 2010). The literature on the external governance of the EU’s migration policy has shed a light on the dynamics and motivations behind EU’s projection of rules and policies (Lavenex, 2010) often portraying the EU as a homogeneous and rational actor, unilaterally exporting its “European interest”.

Instead of reinforcing the perception of third countries as simple receiving units of European policies, we choose to focus on the agency of the local actors and the feedback effects involved in the negotiations for the implementation of migration policies, that may lead, or not, to a translation of the European objectives into the registries of practices of the partner countries. Inspired by the work of researchers focusing on the neighbourhood of the European Union in the study of the its migration policy regime, such as El Qadim (2015), Michalon (2015, 2009, 2007) and Boubakri (2016, 2013, 2008), we have adopted an angle of analysis that moves away from a Eurocentric approach and that instead perceives the external dimension of EU’s migration policy as “a conjunction of the ideas formulated by the actors from the member states and the European agencies, and that takes into account the claims of the third countries”. (El-Qadim, 2015, p. 311) Based on this postulate, our research raises the following question: What is the role of the instrument of the Mobility Partnership in the circulation of norms, ideas and practices related to the “good governance” of international migrations and are these internalized by the partner countries?

In order to answer our research questions, we will present the results of a comparative analysis of selected countries of the Eastern neighbourhood benefitting from a Mobility Partnership. We have adopted an “instrument approach” to our analysis, inspired by the work of French social scientists Pierre Lascoumes, Patrick Le Galès, Bruno Palier, Sabine Saurugger and Yves Surel. As defined by Le Galès and Lacousmes, a policy instrument is “a device that is both technical and social, that organizes specific social relations between the state and those it is addressed to, according to the representations and meanings it carries” (Lascoumes & Le Galès, 2004, p. 5). Defining the policy instrument as an institution, these authors argue that a device is rarely neutral, but rather is made up of representations of social issues that are diffused through its operationalization. The policy instrument rests on cognitive and normative frameworks, meaning that it determines the “ways actors will behave, creates uncertainties on the effects of power relations, favours certain actors allocating them new resources, while excluding others” (Saurugger & Surel, 2006, p. 201). With this analytical framework in mind, we attempt to understand the dynamics guiding the Mobility Partnership from its negotiation to its implementation and how it participates in the diffusion of ideas and practices related to the management of migration, linking this approach with the literature on Europeanization.

In order to maintain our “decentralized” angle to the analysis of the implementation of a European policy instrument of “soft law” in third countries, the definitions of Europeanization addressing the cognitive component of this process are best suited to our research. Most scholars have defined Europeanization as the institutionalization at the domestic level of European policies and politics (Featherstone & Radaelli, 2003, p. 30). In that line of thoughts, Europeanization can be understood as a process through which objectives, methods, rules and instruments, to ways of doings, shared beliefs and values are assimilated at the local level. Following Baisnée and Pasquier’s idea of using Europeanization as a working tool, we propose to look at the Mobility Partnership as a vector for the Europeanization of third country’s migration policies. Even if the Mobility Partnership is an instrument of soft power, without legally binding constraints, it still serves as a vehicle for diffusing a certain vision of the world and can transform the knowledge and the know-how of local actors based on the European game (Baisnée & Pasquier, 2007, p. 218). Most scholars working on the Mobility Partnerships have demonstrated its persistence in pursuing the traditional security-oriented objectives of the EU in third countries (Carrera & Hernandez i Sagrera, 2009). We hope to add a layer of analysis into this field of study by observing the pedagogical capacity of the Mobility Partnerships (Kunz & Maisenbacher, 2013) in orienting the Europeanization of the migration policies in third countries.

According to Lascoumes and Le Galès, the research design in the analysis of a policy instrument needs to retrace its history, unveil its normative and cognitive frameworks, look into the network of actors it contributes to put in place and the effects it produces (Lascoumes & Le Galès, 2004, pp. 363-364) Through field studies in Moldova and Georgia, countries considered by the European Commission as the “best pupils” in the implementation of their Mobility Partnerships, we interviewed the institutional and non-institutional actors involved in these Mobility Partnerships, with the ambition to interrogate whether this instrument leads to a “common understanding” between the EU and the national actors, that would translate in the “formulation of policies and conformation of practices in the field of migrations” (Channac, 2006, p. 400). Sixty-one qualitative interviews were conducted with the actors susceptible to cover the entire field of actions of the Mobility Partnerships for these study-cases. The following table shows the repartition of the interviews conducted across countries and the occupation of the interviewees:

**Table:** 

Role of actors by country	Moldova	Georgia	Brussels	Total
National governmental institutions	9	8		17
Local project coordinators - civil society	8	5		13
European Delegations - Policy Officers	2	1		3
European Commission - Policy Officers			6	6
Local representations of Member States	2	1		3
European Experts	4			4
International Organizations	6	7	2	15

Thus, we will first retrace the origins of this policy instrument in the context of the evolution of the external dimension of the EU’s migration policy and in the local context of our two main study-cases, Moldova and Georgia. We will then discuss our findings based on our interviews with the “intermediate” actors responsible for the negotiation and the implementation of the Mobility Partnerships.

## Tracing the origins of the mobility partnerships

Since the reformulation of the Global Approach to Migration in 2011, the focus of the external dimension of the European migration policy has strategically moved from the Eastern border to the South of the Mediterranean (European Commission, 2011) and the Mobility Partnership seems to have gained in importance to become a key mechanism of cooperation for the European Commission. Tracing the origins of this political instrument allows us to grasp the representations, values and principles at the heart of its foundation, marking a rupture from the previous measures undertaken by the EU to deal with this social issue.

### The emergence of the need for a balanced approach to migration management

From the Tampere Summit held in 1999, the government heads of the EU recognized the importance of cooperating through partnerships with third countries in order to achieve their migration control objectives (Tampere European Council, Presidency Conclusions, 1999). Migration control objectives increasingly became intertwined to the larger framework of cooperation with the EU and appeared in economic agreements as well as in the field of development aid. Developing migrants’ countries of origin is increasingly understood as providing an alternative to emigration and the European Commission has since 1994 pushed for enhanced synergies between migration and development with the aim to reduce migratory pressures (European Commission, 2002, p. 7). The global discourse on migration within the sphere of the European Union is gradually changing, acknowledging the failures of previous restrictive policies. As stated by the European Commission in a communication from 2000: “it is clear from an analysis of the economic and demographic context of the Union and of countries of origin, that there is a growing recognition that the “zero” immigration policies of the past 30 years are no longer appropriate (…). In this situation, a choice must be made between maintaining the view that the Union can continue to resist migratory pressures and accepting that immigration will continue and should be properly regulated and working together to try to maximise its positive effects on the Union, for the migrants themselves and for the countries of origin” (European Commission, 2000, p. 3).

Even though restrictive policies of migration control remain dominant in the external dimension of the European migration policy, those new principles emerged, guiding its evolution: migration can and must be managed to profit the EU, the countries of origin and the migrants themselves, cooperation with third countries is essential to reduce migratory pressures and development in the migrants’ countries of origin can provide an alternative to migration. The following cooperation frameworks proposed by the EU reflect this new posture and impose these principles as self-evidence. With the objective to develop closer relations with its neighbours following the 2004 enlargement, the EU launched the European Neighbourhood Policy (European Commission, 2003), which further contributed to the transfer of the European “best practices” in the field of migration management. Paradoxically, the evolution of the cooperation between the EU and third countries in the field of migration also seems to indicate the increasing role attributed to third countries in the formation and consolidation of the internal security strategy of the Union (Gabrielli, 2007; Guiraudon, 2010).

The highly mediatized events of Ceuta and Melilla that occurred in 2005, along with the proliferation of international forums addressing through a new angle migration-related issues (the Bern Initiative and the United Nations Global Commission on International Migration, among others) prompted the European heads of government to convene and propose an alternative solution to the management of migration. Following the European Council in Hampton Court in October 2005 (Brussels European Council, Presidency Conclusions, 2005), the guidelines for a “Global Approach to Migration” were laid.

Aspiring to move away from the previous security-oriented approach to the management of migration, unpopular with the countries of origin, this new political framework aims to equilibrate the relationship with the partner third countries through new instruments of cooperation such as the Mobility Partnership. At the heart of this approach is the willingness to provide “winning” solutions to all parties involved in the migration phenomenon. Divided into three, then later four pillars, the Mobility Partnership is expected to evenly address the facilitation of legal migration unto the territory of the EU, the enhancement of the link between migration and development, the fight against irregular migration and the international protection of migrants. Hence, the political instrument of the Mobility Partnership surfaces within the context of emerging global discourses on migration management and on the migration and development nexus. The usage of the term “partnership” to refer to the cooperation with third countries emphasizes the engagement to be taken by the parties involved to share the responsibilities related to an “efficient” governance of migration and the willingness of the EU to work collaboratively with third countries. The determination of the pillar structure of the Mobility Partnership translates into actions the ascending discourses of the EU on the management of migration flows: mobility must be facilitated for certain categories of individuals, while migration and border control must be reinforced to prevent unwanted migrants to reach Europe; development cooperation should be enhanced to refrain potential migrants from leaving their home countries and the national protection systems in third countries need to be consolidated so that asylum-seekers can obtain the refugee status in the neighbouring countries of the EU.

The introduction of the Mobility Partnership in the external dimension of EU’s migration policy corresponds to the three reasons enumerated by Lascoumes and Le Galès on their analysis of innovation in policy instruments (Lascoumes & Le Galès, 2004, p. 358). A new instrument is a political act that signals both a rupture with the anterior actions and a quest for efficiency. Additionally, new instruments carry values meant to renew public action. In the context of the Mobility Partnership, its introduction comes at a time when the European Union realized that its previous measures aiming to stop migration flows were not only inefficient, but also heavily criticized. With the launch of the Global Approach to Migration, considering migration as a multifaceted phenomenon that must be addressed with a balanced approach, the EU adopted a novel posture that differs significantly from its previous stance on migration. The Mobility Partnership was the instrument thought to contribute to a paradigmatic change that would (re-) produce representations and meanings related to migration, thus operationalizing the “theory” behind the Global Approach to Migration.

### The third country selection process for the mobility partnership

The first communications of the European Commission on the Global Approach to Migration in 2007 prioritized Africa and the region of the Southern Mediterranean as the geographical zone where the attention and the first actions should be directed, perceived by the member states as more “problematic”. Nonetheless, the Working group on Migration and Asylum first selected countries from the Eastern Neighbourhood to participate in the first generation of Mobility Partnerships (Interview Policy Officer DG-Home, 2016). The intention behind this geographical redirection of attention was to experiment this new policy instrument on countries that did not present significant migration-related issues for the member states, in order to exploit the demonstrative potential of the Mobility Partnership, and use it on future negotiations with countries coinciding more closely with their strategic interests.

The Mobility Partnership is, in the case of the Eastern neighbours, the first instrument of cooperation proposed by the EU to deal exclusively with migration and border issues, placing for the first time these questions on their national political agenda. The previous forms of cooperation designed for the Post-Soviet countries, the TACIS (Technical Assistance to the Commonwealth of Independent States) program and the Partnership and Cooperation Agreements were multidimensional, with initiatives thought to facilitate the transition towards a democratic regime, the state of law and a market-based economy. The Mobility Partnership thus represents the first bilateral form of cooperation on migration for these countries, most of which did not beneficiate from any bilateral relations with member states on this issue.

In order to be considered for a Mobility Partnership, the candidate third country must consent to a number of engagements, including its willingness to open negotiations for an EU readmission agreement, to fight irregular migration, to cooperate with Frontex, to exchange information with the competent authorities of the member states and to encourage return and reintegration of their emigrants (European Commission, 2007). In return, the European Commission and the member states agree to *consider* legal migration possibilities, to assist third countries by building their capacities to manage migration flows, to encourage measures to counteract brain drain and finally, to facilitate the procedures for the Schengen visas. Even if the Mobility Partnership is presented as a legally non-binding declaration, with an informal and flexible framework, the negotiations for the conclusion of this instrument rest on a top-down approach, meaning that potential partner countries must first agree to certain engagements mostly related to the fight against irregular migration in order to be considered as a partner for cooperation.

Strongly encouraged by the local branch of the International Organization for Migration (IOM) (Interview Moldovan Ministry of Foreign Affairs, 2016), the Moldovan authorities seized the opportunity to open a dialogue with the EU on the conclusion of a Mobility Partnership, and submitted proposals for cooperation through a series of “non-papers”. The Moldovan propositions insisted on their engagement to reduce the number of emigrants traveling towards the EU and to collaborate alongside the EU and its agencies on the fight against irregular migration and border management, corresponding to the will of the member states and the European Commission. The national authorities admitted not being in a position enabling them to formulate their cooperation requirements since they were mostly satisfied with the opening of an exclusive dialogue with the EU. Georgia quickly followed Moldova’s example and launched the negotiations for a Mobility Partnership shortly after the end of the war with Russia in August 2008. The conclusion of this Mobility Partnership was interpreted in this context as a form of support from the European Community to this small nation threatened by a powerful neighbour. In this case, the Office of the State Minister for European and Euro-Atlantic Integration of the Georgian government led the negotiations. While the mandate of this political body is to pave the way for an ever-increasing integration into the EU, it clearly is not concerned with the migration-related issues facing Georgia.

The selection process of a partner country for the conclusion of a Mobility Partnership demonstrates the asymmetry inherent in this instrument from its beginning. Moldova and Georgia, both inexperienced in migration management, willing to enforce migration control objectives and prioritizing closer relations with the EU in their respective political agendas, agreed on the terms of the Mobility Partnership, without much negotiation. Unlike countries of the Southern neighbourhood, familiar with bilateral agreements concluded with different EU member states to cooperate in the field of migrations, Moldova and Georgia, driven by their European aspirations, took on the role of subordinates in this new form of cooperation, requiring a certain number of constraining engagements on their behalf.

### Defining the content of the mobility partnership

The communications of the European Commission on the Mobility Partnerships have done little to demystify what the latter would contain. At the time of its first implementation, the Mobility Partnership still appeared as a nebulous instrument to all participating parties, from the member states to the partner country, and even to the Policy Officers from the European Commission themselves (Interview Policy Officers, DG Home Affairs, 2012-2016). A European team of experts is deployed in the partner country to provide recommendations that will, along with the stated priorities of the local authorities, form the content of the Mobility Partnership. In the case of Moldova, the national authorities formulated the return of their nationals and their subsequent economic and social reintegration as their main priorities. For Georgia, the reintegration of their nationals and the management of their borders were stated as the main objectives to be achieved. Both countries’ priorities coincided largely with the interests of the participating member states. The institutional actors in charge of the negotiations of the Mobility Partnership on the Moldovan side admitted being fully conscious that the objectives that landed on their partnership reflected more closely the priorities wanted by the EU, but did not perceive it as an anomaly since it proved their commitment to adhere to EU rules.

Furthermore, it is worth noting that in all of the Mobility Partnerships with the countries of the Eastern Neighbourhood, the initial projects were elaborated without a clear understanding of the migratory situation, since no reliable statistical data or researches were available. Rather, general principles on migration guided the design of the projects, considering migrants in that sense as a homogeneous group without particularities, presuming that successful initiatives from different areas of the world could simply be geographically transposed and obtain similar results. The projects emerging from Mobility Partnerships correspond inevitably to the political will of the participating member states, as they will only contribute, financially and operationally, to the projects reflective of their national imperatives. Without clear membership perspective for either country, the promise of visa-free travel to the EU for Georgian and Moldovan nationals, holders of biometric passports, has been the strongest incentive to proceed with the implementation of the measures under the Mobility Partnership (Interviews with institutional actors Moldova and Georgia, 2014-2016).

It is under the framework of the Mobility Partnership that countries like Georgia and Moldova have started coordinating their actions in the field of migration management, by reuniting on a regular basis all of the actors involved at the national level to collectively debate about migration-related issues, accompanied by representatives from the European Commission, the member states and from selected International Organizations. The European Commission has insisted for the inclusion of local civil society organizations in these regular coordination meetings and has encouraged them to join in the national efforts by providing substantial funding for their activities, which has, at times, turned them into valuable sources of expertise for the national authorities. The creation of “local platforms of cooperation” in the case of Moldova or of a State Commission on migration issues in Georgia have played a crucial role in the socialization of the actors relevant to the field of migration in each country and have formed closely-knitted networks that now share common vocabulary and conceptions on the phenomenon of migration. It is within these circles that new priorities for future cooperation are elaborated and draft laws discussed. Our observation of meetings held under these frameworks revealed that English is often the language of communication and that the national institutional actors have come to master the formulas related to the “global migration governance” discourse, rarely interrogating their foundations. Certain institutions have taken a prominent role (Ministry of Foreign Affairs, Ministry of Interior, Ministry of Justice in the case of Georgia) while others appear excluded from the process. Surprisingly, it is often the state agencies working specifically on migration related issues that seemed left out from these networks.

The pilot Mobility Partnerships concluded with the countries of the Eastern Neighbourhood ambitioned to exploit the demonstrative potential of this new instrument. The EU has played a significant role in setting migration-related issues onto the national political agendas of the countries of the Eastern Neighbourhood. For Moldova and Georgia, the cooperation with the EU in the field of migration management, although asymmetrical, corresponds to their objective of facilitating the access to the territory of the EU for their citizens and more globally, to their aspirations of eventually integrating the Union. Vertical and horizontal processes of Europeanization appear to be intertwined in the implementation of the Mobility Partnerships. Although it remains a non-binding political instrument, it follows a top-down approach by constraining the partner third countries to accept a number of engagements in order to advance in the cooperation proposed by the EU. On the other hand, the socialization of the actors through the setting of cooperation platforms involving representatives from the national authorities as well as from the European Commission, the member states and the International Organizations creates networks that facilitate the learning processes and the dissemination of principles, norms and practices related to the “good” governance of migration.

## Beyond the discourse, an overview of the implemented initiatives

Having looked at the way the first Mobility Partnerships were elaborated and with the objective to interrogate how the guiding principles on migration management of the Global Approach to Migration are diffused through this instrument, we now propose to take a deeper look at the implementation of the initiatives falling under this framework of cooperation. Through the interviews conducted with the relevant actors, along with the examination of the “scoreboards” detailing the projects for each partner country, we observe an unequal repartition of the efforts through the pillar structure of the Mobility Partnerships.

### A mix of horizontal and vertical processes of Europeanization to transform the management of migration in third countries

The model of migration control proposed by the EU through the Mobility Partnership to the Eastern Neighbourhood countries coincides for the most part with their stated national interests (Interviews with Georgian public servants, 2014). Before the conclusion of Mobility Partnerships, Moldova and Georgia did not have clearly defined national migration strategies or policies. Under the Saakhashvili government (2004-2013), Georgia had a *“*laissez-faire*”* approach to the management of migration that, in its view, was beneficial for its tourism industry and to attract foreign investors. Citizens from 118 countries could enter Georgia without visas, stay up to 360 days, find employment without work permits, and leave the country for a day in order to renew their stay (Chumburidze et al., 2015). The European Commission, along with the local branch of the IOM, pressured the Georgian government to review its migration-related policies. Georgia only adopted its first migration policy in 2013 (Law on the Legal Status of Aliens and Stateless Persons) (Makaryan, 2013), reducing the list of countries exempted of visas and allowing foreigners to stay 90 out of 180 days, in order to be in line with the requirements of the Visa liberalization action plan. It was reported that following the introduction of this law, tourism went down by over 40,000 arrivals within a few months, prompting the chairman of the International Chamber of Commerce of Georgia to call for a review of this legislation “poisoning the life of foreigners and affecting investors’ confidence” (Financial Times, March 26th 2015).

Meanwhile, the emigration of Moldovan nationals, estimated by the IOM, the World Bank and the National Border Police at about a quarter of its work force, was not given much attention by the State administrations. It is through the instrument of the Mobility Partnership that migration policies were structured in both of our case studies. Looking at the national migration strategies for both countries for the current period, the influence of the European Union is obvious and the priorities enumerated in each document reflect the framework of the Global Approach to Migration. In both papers’ introduction, it is stated that the approximation of their policies to the EU norms and standards in the field is in line with their objective of eventually integrating the Union and the priorities are often closely linked to current projects funded by the EU. However, as argued by Makaryan (2013), “borrowing” elements from the EU (mostly immigrant-receiving countries), can lead to a disconnection with the reality of these countries (emigrant-sending). The driving principles of the Global Approach to Migration are diffused in these contexts as norms to be adopted, “understood as a standard of appropriate behaviour for actors” (Trauner & Wolff, 2014, p. 15).

In both of our study-cases, legislation on migration was first introduced through the cooperation platforms with the EU and with the help of international organizations such as the IOM and the UNHCR. Local branches of international organizations and European experts are invited to participate in the working groups responsible for elaborating new laws and are quite active in the process, sometimes even proposing the first draft. High-level European experts are often deployed for long-term missions in various national ministries to provide their assistance in reforms and in the formulation of new laws, as it has been the case with the Bureau for Migration and Asylum in Moldova and in the Ministry of Labour, Health and Social Affairs in Georgia. At times, the delays for a new law on migration are imposed by the European Commission, which may lead to the adoption of fast-track legislation that may require amendments at a later time. As expressed by a Moldovan civil servant interviewed: “*Most of the time, we use French legislation which was also put in practice in Romania, and for us, with the language facility, we are very inspired by French or Romanian legislation, even if they are not adjusted to the reforms we just accepted and because of deadlines from the EU, we are afraid not to please the European Commission…”* (Moldovan public servant, 2012).

We must underline the role of “driving belt” played by the local branches of international organizations in the implementation of the Mobility Partnerships. Perceived as a “necessary tactical step providing more legitimacy to EU’s demands and actions in relation with its partners” (Hernandez i Sagrera & Korneev, 2012, p. 9), international organizations often act as intermediary between the EU and the partner countries, providing their expertise and know-how to the inexperienced state institutions responsible for implementing elements of the Mobility Partnership. Essential agents of the diffusion of the “global migration governance” discourse, they can be both “executers” of the European migration policy, while at the same time, active in the representation of the partner country (El Qadim, 2015, p. 294). Because of their privileged position on the field and with Brussels, they play an active part in the socialization processes of the local actors part of the network of migration governance and in the Europeanization of norms and standards related to migration management. Most of the larger scale projects falling under both Mobility Partnerships have been implemented by an international organization with the help of member states and the relevant national authorities. If we look at the case of the IOM in Moldova, their involvement in the Mobility partnership goes back to the negotiation phase, where they assisted the Moldovan government in formulating their priorities for cooperation. Consequently, they have supported both the European Commission and the local authorities in designing projects that would fall under this new framework of cooperation and were then in a privileged position to successfully answer the calls for projects. Working closely with the national authorities, the local branches of the ICMPD and the IOM diffuse beliefs, principles and values related to migration management through the implementation of projects related to mainstreaming migration into development, reinforcing migration and border controls, building the capacities of state authorities and facilitating the return and reintegration of migrants into their home countries. Their influence in the circulation of norms and practices related to the perception of efficient migration management is undeniable in both contexts and has contributed to the development of a “common understanding” on those issues among the local actors.

Adopting a restrictive posture on immigration was, in both of the case studies, a highly popular alternative to the vacuity that previously existed, since it demonstrated that the national authorities were conforming to European norms of migration control, that they “cared” about their nationals migrating abroad and appeased the public opinion, worried about potential influx of foreigners onto their territory (Interviews with civil servants in Moldova and Georgia, 2014-2016). Numerous local political discourses concur with this idea, insisting that the modernization of their country will happen through the European model, rather than the Russian alternative, and that by doing so, will become increasingly attractive destination or transit states for potential migrants. In that line of thoughts, adopting restrictive measures on immigration is necessary to proactively address this eventuality (Wunderlich, 2013). This “tough” stance on migration from the Eastern partners can be found in the cooperation on border-related issues and readmission. The EU has engaged in considerable efforts in the region to consolidate the capacities of their border regime, supporting a border assistance mission between Ukraine and Moldova,^1^ as well as the border authorities in the South Caucasus.^2^ These measures are appreciated by the partner countries, hoping to strengthen their position from their threatening neighbours,^3^ but can also be seen as a great help for countries like Georgia, in their route toward NATO accession. By perpetuating discourses now internalized on the need to reinforce border and migration control with the assistance of the EU, national authorities are signalling that they share common issues with the European political community and that they are conforming to the dominant scheme of thoughts on the issue.

Thus, it is with no surprise that the initiatives that received the most attention so far under the Mobility Partnership for both countries are those centred on the fight against irregular migration and border management. The willingness of the partner countries to adopt migration control measures, led by the belief that their rapprochement with the EU will inevitably lead to an increase number of immigrants into their land, coincides with the strategic interests of the EU in its cooperation with third countries.

The external action of the EU functions under the principle of positive conditionality, meaning that more reforms done by the governments of partner countries to transform their management of migration to the model of EU’s best practices will lead to more funds, to a closer cooperation with the EU, to a visa liberalization action plan or to an Association Agreement.^4^ Although the Mobility Partnership is portrayed as a non-binding and flexible instrument, the principle of positive conditionality seems to have played a significant role in the parallel opening of the dialogues on visa liberalization in both cases. According to a policy officer of the DG HOME that we interviewed, the Mobility Partnership is *“a framework for political dialogue and cooperation. And within this frame, in the area, it worked really well, they have many more things happening in the area than before.”* To understand how this instrument of soft power obeys to the principle of positive conditionality, he adds: “*And also in the case of Moldova, it’s been quite important in the context of visa dialogue, because by engaging this Mobility Partnership and starting this sort of regular dialogue and coordination, there was sort of trust built, which made it easier to engage on the visa dialogue when it came time to”.* Through the use of positive conditionality, the Mobility Partnership can be perceived as a soft power policy instrument preparing the field for the conclusion of more legally-binding agreements. Hence, the Mobility Partnership can be used as a stepping-stone by partner countries to engage in deeper cooperation frameworks with the EU, while allowing the European interests to descend in their registries of practices through mechanisms of positive conditionality. In that context, the political instrument of the Mobility Partnership did not receive much resistance in the implementation of initiatives falling under the migration control pillar of its structure since both Georgia and Moldova were keen on adopting reforms in that domain, while at the same time seizing the opportunity offered by the European Union for increased cooperation.

### What about the mobility Partnership’s “winning” solutions for migrants?

Only one complete evaluation of the Mobility Partnership has so far been conducted. Initiated by the IOM, the European Commission and the government of Moldova, the 2012 evaluation of the Moldovan Mobility Partnership revealed some of its lacunae as perceived by the stakeholders. The facilitation of legal migration and the international protection pillars of the Mobility Partnership received insufficient attention according to those surveyed. Moldovans admitted being disappointed by the limited mobility opportunities offered to their nationals. Just by its title, the “Mobility” Partnership holds the premise of offering greater mobility perspectives to the nationals of the participating third countries. Even though the concept of circular migration appears as a top priority from the partner countries in all of the Mobility Partnerships, the member states have been reticent, to say the least, to propose such schemes. Taking a closer look at the projects implemented in both the Moldovan and the Georgian Mobility Partnerships, one can notice the broad and vague meaning of the “facilitation of legal migration and mobility” category under which they fall. Most of their objectives are focused towards the return of migrants and their social and economic reintegration, the reinforcement of the national employment services capacities and the diffusion of information on the possibilities to legally migrate to the EU and of the risks of irregular migration. It appears from the evaluation that Moldovans did not share the same understanding of “mobility” than their European counterparts. In the context of the Mobility Partnership, even though circular migration schemes were initially identified as one of the benefits from this cooperation, only the short-term mobility of selected categories of individuals (students, tourists, researchers and business people) has been included in the framework, notably through the visa facilitation agreement linked to the conclusion of the community-wide readmission agreement. The pursuit of the long-term objective of rapprochement to the Union and of the opening of further frameworks of cooperation with the EU have prevented the local authorities from Moldova and Georgia to be more assertive with their demands for short-term mobility opportunities for their nationals through their Mobility Partnership.

As for the international protection pillar, by looking at the matrix of initiatives in all of our study cases, we can only notice the weak support towards this dimension of migration management. In the case of Moldova, there has been so far very few initiatives (four in total led by two member states and ICMPD) aiming to “reinforce the capacities” of the national institutions in their ability to provide protection to asylum-seekers, that mostly took the form of discussions between experts. Through the entire region of the South Caucasus, only one project was directed towards international protection, and consisted in a quality analysis of the national asylum systems for all three countries. The lack of interest from the European Commission and the member states in the international protection pillar contradicts an argument often heard in the literature on the external dimension of EU’s migration policy. Strengthening the asylum systems in the neighbouring countries of the EU should be part of the Union’s strategy to “externalize” its policies of control and should be encouraged in order to reduce the number of individuals seeking asylum in the member states. However, this logic does not seem to be reflected in the countries of the Eastern Neighbourhood, as shown by the weak support to projects related to the international protection pillar of the Mobility Partnerships.

Taking a deeper look into the implementation of the Mobility Partnerships relativizes the discourses conveyed by the European Commission on this policy instrument. Since member states will only financially and operationally participate in the initiatives that correspond to their own interests, the proposed “balanced” structure of the partnership is undermined. When overviewing the implementation of the legal migration and international protection pillars of these policy instruments, one can wonder what place is left for the actual migrants. As coined by Stefan Rother, the Mobility Partnership resembles a case of “global migration governance without migrants” (Rother, 2013), since most of the initiatives are centred on the state and its administration and on diffusing ideas and practices related to the “good” management of migration. To quote a Moldovan public servant interviewed: “*the biggest projects are on the capacity-building with migration management […] nothing with the support going directly to asylum-seekers or to people […]*”.

Interestingly, the migration and development pillar has led to significant developments in both of our study-cases, demonstrating the internalization of the migration and development discourse among the local actors. The migration and development nexus stems from a consensus between policy-makers, consolidated over the years in the numerous trans-governmental forums on migration, that perceives migrants as central actors for the development of their country of origin. Moldova has persistently been depicted as the “good student” in the implementation of the “migration and development” pillar of the Mobility Partnership and as an example to follow for other partner countries. Most of our Moldovan interviewees have enthusiastically recalled that representatives from this country have been invited on numerous occasions to share their experience, notably in the Global Forum on Migration and Development. Most of the initiatives in both of these countries mainly focus on the involvement of the diaspora in the development of the country through support for the creation of start-up businesses, job-matching, skills development, recognition of qualifications and the simplification of procedures for the sending of remittances. The IOM has been mandated to design projects aiming at “strengthening the development component of the Mobility Partnership” in Moldova and Georgia, initiatives that have again mainly consisted in building the capacities of the national policy-makers, in identifying “best practices” and encouraging the mobilization of the diaspora. Attempts to reach out to the diaspora of both countries have taken many forms, including through the importation of the “co-development” concept and through the creation of national agencies responsible for diaspora issues.

The annual participation of Moldova at the Global Forum on Migration and Development and the regular interactions between local actors, international organizations and European experts have facilitated the internalization among the policy-makers of the belief that greater development in the migrants’ countries of origin will reduce emigration and of the migrants’ central role in this dynamic. However, our interviews with the local actors revealed that the difficulties in the implementation of the projects related to migration and development were, according to their opinion, to be attributed to a lack of consideration of the local context, to insufficient attention paid to the needs expressed by the migrants themselves and to frequent misunderstanding with the government and local officials on the objectives of each initiative. As expressed by a Moldovan project officer interviewed: “*How can you say to a migrant who left the country to survive to give money for the reconstruction of a school. I mean this is ridiculous! He’s surviving and thinking about how to save enough to buy a flat when he comes back, pay the studies for his son and now you talk about… I mean for Moldova, it is not feasible yet, the co-development concept […]”.*

The impacts of the Mobility Partnerships’ initiatives are rarely evaluated in the sense of their actual consequences on migrants and/or nationals. Projects are discussed at the local cooperation platforms reuniting the network of governance on migration issues in rather vague terms. Accordingly, their success or failure is not based on factual measurements of their outcomes, but rather seems to be determined by the level of “ideational convergence” (Radaelli & Pasquier, 2008, p. 38) they have led to. The regular interactions among the actors, the implementation of projects designed in Brussels or in the member states of the Union, the presence of European experts in the national ministries, along with the prominent role played by international organizations on the field are all elements contributing to the learning processes of the national authorities on the know-how and the development of knowledge related to the perception of efficient migration management. While we can attest of top-down Europeanization processes through mechanisms of conditionality and through the imposition of reforms related to border and migration management in the partner countries, it also appears that more subtle forms of transfers are at play. Socialization and learning mechanisms are put in place to diffuse ideas, knowledge, norms and practices related to migration and their internalization is made visible through the discourses relayed on those issues by local policy-makers.

## What future for the mobility partnership? Discussion on the outcomes and limits of this instrument

From our fieldwork experience in the Eastern neighbourhood countries that have agreed to sign up for Mobility Partnerships, some conclusions can be drawn on this new form of cooperation. Even though the EU has pursued a combination of traditionally restrictive objectives, the partner countries have demonstrated in their implementation of this policy instrument a capacity of action and have been “able to use European policy as an opportunity rather than responding to a pressure” (Featherstone & Radaelli, 2003, p. 46). The concept of the “usage of Europe” coined by Jacquot and Woll (2003) can help us understand how Europe can represent a resource for local actors, and leads us to question the assumption of a unilateral transfer of norms (Hernandez i Sagrera & Korneev, 2012) that would operate through the implementation of a policy instrument.

### On the positive outcomes of the mobility partnership

Reflecting on the realizations achieved through the Mobility Partnership, we must highlight the role it has played in placing the issue of migration onto the national political agendas of the countries of the Eastern Neighbourhood. According to their own words, the Moldovan and Georgian officials have “come to realize” the amplitude of this phenomenon and the need for action, demonstrating the progressive construction of a “common understanding” on migration issues and how they should be addressed (Channac, 2006, p. 13). In both Moldova and Georgia, the State institutions had until then provided little administrative support to their migrants. The reflection around this topic has led to the formulation of development projects that, for example, addressed the question of the recognition of qualifications acquired abroad, the care for the children and the elderly left behind and the outreach to the diaspora. Numerous studies have been conducted under the framework of the Mobility Partnership, in order to understand the characteristics of the migration flows emerging from this area, to interrogate migrants on their needs and to provide recommendations to the local administrations.

The interviews have revealed that the main reason explaining why migration took such an important place on their respective national political agendas, particularly in Moldova and Georgia, is not because of the gravity of the issues related to this phenomenon nor because of a sudden realization of what must be done to address the concerns of their citizens residing abroad, but because it is one of EU’s top priorities and linked to strong incentives for their respective governments. To quote one of the interviewees from the Georgian civil society: “*People don’t do migration management and control for migration management and control but for visa-free regime and to do good in EU cooperation*”. Unlike the Visa liberalization action plan, the Mobility Partnership, as a non-binding instrument of soft power, does not have an end result that serves as a strong incentive. Rather, it has been perceived by the Georgian and Moldovan authorities as a stepping-stone to engage with the EU on the dialogue for a visa-free regime and for closer cooperation. Nonetheless, even if these strategic interests motivated the Moldovan and Georgian authorities to embark on this new cooperation framework, the preferences of the actors have been gradually transformed according to those of the EU.

For countries like Moldova, presenting a priori little interest for deepened relations with the member states, the Mobility Partnership has represented a truly unique opportunity to gain more visibility and to open a privileged dialogue with the EU. Both countries have achieved one of their top national objectives through their implementation of the Mobility Partnership, the liberalization of the Schengen visa for their citizens. The Mobility Partnerships, entangled in the web of cooperation deployed by the EU and its member states in the region, cannot be analysed separately from the other instruments of cooperation. Nonetheless, its implementation, when deemed satisfactory by the European Commission, can deepen the relations, leading to an ever-increasing approximation of the national policies and practices to the EU norms and standards.

### On the limits of the mobility partnership

The experiences of the Mobility Partnerships in the Eastern neighbourhood present numerous limits. First and foremost, the analysis of the existing partnerships reveals that it is after all an empty shell, and that its content will inevitably depend from the level of interest and engagement of the participating member states, the European Commission and the third country. For those reasons, we can question whether it has been used as an entryway to push for the conclusion of more constraining agreements, such as the community-wide readmission agreements.

Member states have also demonstrated incoherence in their support for the Mobility Partnerships. As an example, even though Georgia’s Mobility Partnership is the one that received the most signatures from member states (16), it did not translate into a strong commitment from them, and very few projects emerged if we compare to Moldova (26 projects in Georgia and over 85 in Moldova). Consequently, 3-day study visits of detention centres in a member state for a handful of civil servants from the partner country could symbolize the engagement of a signatory member state into this instrument of cooperation. This example illustrates in our opinion the gap between the rhetoric surrounding this framework of cooperation, its stated objective of coherence and comprehensive action, and its actual implementation.

Most of the Moldovan and Georgian officials interviewed have agreed on the lack of visibility and comprehension of this instrument, unsure at times of its purpose or added value besides leading to some proximity with the EU and paving the way for the Visa Facilitation and readmission agreements. For those reasons mainly, these countries have been more “reactive” to their Mobility Partnerships, unable to elaborate a long-term strategy of their objectives and priorities under this framework of cooperation. They have also attributed the implementation gaps of the Mobility Partnership to an insufficient consideration of the local context in the conception of the programs of action. Both countries appear after all to have little ownership over their migration policies, mainly influenced by the direction imposed by the EU. Since both countries were previously inexperienced in the field of migration management and acknowledged that migration is a priority issue for the EU in its foreign relations, the governments of Moldova and Georgia have welcomed the opportunity represented by the Mobility Partnership, and have strived to perform with this policy instrument.

In the end, implementation gaps reveal the lack of attention paid by the initiators of the projects to their outcomes and the lack of comprehension of the local context when designing initiatives. The consequences of the Mobility Partnerships’ projects are rarely at the centre of the discussions during the local cooperation platforms since the focus is usually on the dissemination of knowledge and “good practices” in handling the phenomenon of migration. Intersecting mechanisms of Europeanization are involved through the implementation of the Mobility Partnership. Cognitive Europeanization is achieved through socialization and the learning of new knowledge made possible through the network of governance set up by this political instrument. At the same time, more coercive measures are taken by the EU to ensure that the national legislation of the partner countries is aligned to its *acquis* and border and migration control objectives are conform to the European interests. In this sense, our overview of the political instrument of the Mobility Partnership demonstrates, as Lascoumes and Le Galès have stated, that, far from being neutral, it serves as a vehicle for the dissemination of meanings, representations, ideas and values that lead to a convergence in the ways to apprehend the phenomenon of international migrations.

## Conclusion

Overall, the cooperation initiatives launched by the EU following the change in the rhetoric on the management of migration have mainly attempted to proceed to a “system-export of the Area of Freedom, Security and Justice governance mechanisms” (Longo, 2011) through the transfer of knowledge and best practices, and has rarely diverted from the traditional security-oriented logic of action. The principle of conditionality is fundamental, ensuring that even with soft-power instruments, the question of migration is addressed in order to unlock further dialogues related to trade, development and so forth.

Despite the European Commission’s cooperation narrative found in the guiding principles of the Global Approach to Migration, the analysis of the existing Mobility Partnerships challenges the stated ambition of a balanced pillar structure. Instead, few and vaguely formulated development objectives and legal migration possibilities have emerged and appear diluted when compared to the control-oriented measures. This reality exposes the gaps that subsist between the hegemonic security-oriented vision shared by the authors of this policy and the illusion of a transparent and harmonious collaboration proposed to third countries. Through its implementation, the Mobility Partnership implies an exercise of socialization between local actors and European experts, “contributing to the diffusion of knowledge and technologies related to migration management into the registries of practices of the partner countries.” (Featherstone & Radaelli, 2003, p. 46), that has been conducive to the internalization of the “global migration governance” discourse at the local level. The national migration strategies of both countries, with principles closely similar to those of the Global Approach to Migration of the EU, reflect the “accepted and normalized way of thinking and acting about international migration” (Kunz & Maisenbacher, 2013, p. 207) that has emerged over these issues. In that sense, it appears that the Mobility Partnership, rather than bringing solutions to existing problems, participates instead in the construction of representations on a particular issue, in this case, the “efficient” management of the migration phenomenon.

Altogether, the interviews with the Moldovan and Georgian actors of the Mobility Partnership have revealed the progressive appropriation of a European belief system related to the good governance of migration through processes of Europeanization and have also informed of the strategic use that partner third countries can pull from this cooperation to pursue their own political objectives of getting closer to the economic and political community of the Union. In the context of these former communist states, the model of governance proposed by the EU is perceived as legitimate and as argued by Delpeuch, has great chance of being imitated “even with a mythologized and a vague understanding of the practice taken for model and of the added value its importation is susceptible to bring to their activity” (Delpeuch, 2008, p. 12). The Moldovan and Georgian governments have transformed their legislative framework in order to approximate their laws in the field of migration to those of the European Union and have adopted into their registries of practices the measures of migration-control requested by the EU through its various policy instruments.

Although we argue against the unilateral transfer of norms often assumed in the literature on Europeanization and in the study of the external dimension of the EU (Hernandez i Sagrera & Korneev, 2012), both Moldova and Georgia have made a “usage of Europe” that coincide with the interests of the Union. Furthermore, the approximation to EU norms and standards has a “moral background” (El Qadim, 2015) that serves to reinforce the standing of both countries in the international community, allowing Moldova and Georgia to claim: “I am one of you” (Makaryan, 2013b). Even though the implementation of the Mobility Partnerships has at times been a complex task, with local implementers unsure about the purpose of the projects and their coherence with the local reality, in both cases, the European Commission perceives them as achievements, even if no proper evaluation mechanisms have been set to monitor their consequences. Surprisingly, the results from the ‘“pilot” Mobility Partnerships concluded with the countries from the Eastern Neighbourhood have not been highlighted in order to learn lessons from these past experiences and share information on their successes and failures, from the viewpoint of the local actors. It is with the objective to fill this informational vacuum that we have attempted to summarize the outcomes of the Mobility Partnerships concluded with the Republic of Moldova and Georgia.

## Abbreviations

DG: Directorate-General (European Commission)
EU: European Union
Frontex: European Border and Coast Guard Agency
GAM: Global Approach to Migration
GAMM: Global Approach to Migration and Mobility
ICMPD: International Centre for Migration Policy Development
IOM: International Organization for Migration
NATO: North Atlantic Treaty Organization
TACIS: Technical Assistance to the Commonwealth of Independent States
UNDP: United Nations Development Programme
UNHCR: United Nations High Commissioner for Refugees

## Footnotes

1 The European Union Border Assistance Mission to Moldova and Ukraine was launched in 2005, following the request of the Moldovan and Ukrainian authorities to the EU and the signature of a « Memorandum of Understanding » between the three parties. It is a technical and consultative mission, whose principal objective is to improve the capacities of the border services from both countries. The mission also provides guidance for the settlement of the Transnistrian conflict, in line with the European Neighbourhood Policy’s objectives. According to the EUBAM official webpage, it counts 196 agents, from which 80 are experts from 13 Member States of the EU.

2 There is a myriad of projects related to border management financed by the EU in the region implemented through different policy instruments, such as the « Support to the Integrated Border Management Systems in the South Caucasus », « Reinforcing the Capacities of the government of Georgia on Border and Migration Management » and the « Eastern Partnership Pilot Project on better coordination of the protection of land border between Azerbaijan and Georgia » initiatives. The Frontex agency also manages the targeted project of the Eastern Partnership on Integrated Border Management Capacity Building in the region.

3 There are numerous territorial conflicts in the region – Moldova-Transnistria, Georgia-Russia, Armenia-Azerbaijan and Armenia-Turkey, impairing the process of border demarcation between the countries.

4 Moldova and Georgia both signed their respective Association Agreement with the EU in June 2014.
